# Occurrence and multilocus genotyping of *Giardia duodenalis* in black-boned sheep and goats in southwestern China

**DOI:** 10.1186/s13071-019-3367-1

**Published:** 2019-03-13

**Authors:** Dan Chen, Yang Zou, Zhao Li, Sha-Sha Wang, Shi-Chen Xie, Lian-Qin Shi, Feng-Cai Zou, Jian-Fa Yang, Guang-Hui Zhao, Xing-Quan Zhu

**Affiliations:** 1 State Key Laboratory of Veterinary Etiological Biology, Key Laboratory of Veterinary Parasitology of Gansu Province, Lanzhou Veterinary Research Institute, Chinese Academy of Agricultural Sciences, Lanzhou, 730046 Gansu People’s Republic of China; 2 College of Veterinary Medicine, Northwest A&F University, Yangling, 712100 Shaanxi People’s Republic of China; 3 Key Laboratory of Veterinary Public Health of Higher Education of Yunnan Province, College of Veterinary Medicine, Yunnan Agricultural University, Kunming, 650201 Yunnan People’s Republic of China; 4 Jiangsu Co-innovation Center for the Prevention and Control of Important Animal Infectious Diseases and Zoonoses, Yangzhou University College of Veterinary Medicine, Yangzhou, 225009 Jiangsu People’s Republic of China

**Keywords:** *Giardia duodenalis*, Prevalence, Multilocus genotypes (MLGs), Black-boned sheep and goats, Yunnan Province, China

## Abstract

**Background:**

*Giardia duodenalis* is an important intestinal protozoan infecting both humans and animals, causing significant public health concern and immeasurable economic losses to animal husbandry. Sheep and goats have been reported as common reservoirs of *G. duodenalis*, but only a limited amount of information is available for native breeds of these small ruminants in China. The present study investigated the prevalence and multilocus genotypes of *G. duodenalis* in black-boned sheep and goats, two important native breeds in Yunnan Province, southwestern China.

**Methods:**

Fecal samples were collected from 336 black-boned goats and 325 black-boned sheep from five counties (Meishui, Shanshu, Shilin, Yongsheng and Nanping) of Yunnan Province and the genomic DNA was extracted from these feces. The prevalence of *G. duodenalis* was determined by the nested PCR targeting the β-giardin (*bg*) gene. The assemblages and multilocus genotypes (MLGs) were investigated based on analyses of three genetic loci, i.e. *bg*, glutamate dehydrogenase (*gdh*) and triosephosphate isomerase (*tpi*).

**Results:**

*Giardia duodenalis* infection was detected in both black-boned sheep and goats, and the prevalence of *G. duodenalis* in black-boned sheep (21.8%, 71/325) was significantly higher (*χ*^2^ = 36.63, *df* = 1, *P* < 0.001) than that in black-boned goats (4.8%, 16/336). Significant differences in prevalence were also observed in goats and sheep from different counties (*χ*^2^ = 39.83, *df* = 4, *P* < 0.001) and age groups (*χ*^2^ = 97.33, *df* = 3, *P* < 0.001). Zoonotic assemblage A and animal-specific assemblage E were identified in both black-boned sheep and goats with the latter as the predominant assemblage. Based on sequences obtained from the three genetic loci (*bg*, *gdh* and *tpi*), 16 MLGs were obtained in black-boned sheep and goats, including 15 MLGs in assemblage E and one MLG in assemblage A.

**Conclusions:**

Our results not only extended the host range of *G. duodenalis*, but also revealed high genetic variations in *G. duodenalis* assemblages. The findings of the present study also provide baseline data for preventing and controlling *G. duodenalis* infection in black-boned sheep and goats in Yunnan Province.

## Background

*Giardia duodenalis* (syns *Giardia lamblia*, *Giardia intestinalis*) is one of the most common parasites that causes intestinal infections of humans and various animals worldwide [1–3], and about 280 million people have symptomatic infections annually [4, 5]. The prevalence of *G. duodenalis* in humans in developing countries is much higher than that in developed countries [6, 7]. In China, the average prevalence of *G. duodenalis* in humans was 0.85% from 2000 to 2017 (197/23,098) [3]; however, the infection rate is underestimated because people in many areas with poor medical care have not yet been assessed. Giardiasis, caused by *G. duodenalis*, is characterized by diarrhoea, abdominal pain, bloating, malabsorption and weight loss in symptomatic infections [1, 8, 9]. Moreover, diarrhoeal disease, causing about 800 000 fatalities worldwide per year, is the main cause of illness and death for children during the first five years of life [4, 10]; *G. duodenalis* is the second-most common pathogen detected in diarrhoeal stools of children between 12–24 months of age [7]. However, due to drug resistance and known cases of treatment failure [11, 12], it is essential to find the source of the infection for effective control of this parasitic disease.

Thus far, at least eight genetically distinct assemblages (A-H) of *G. duodenalis* have been identified using molecular biological analysis. Among them, assemblages A and B are believed to have zoonotic potential for their broad host range, and assemblage A is more frequently found in livestock than assemblage B, while the remaining assemblages (C-H) are animal-specific groups [1, 13, 14]. To better assess the zoonotic transmission of giardiasis and differentiate mixed infections of assemblages, high-resolution multilocus genotyping analysis using more intra-assemblage variation genes, including β-giardin (*bg*), glutamate dehydrogenase (*gdh*) and triosephosphate isomerase (*tpi*) genes, has been widely used to characterize *G. duodenalis* isolates from humans and animals [1, 15].

Sheep and goats are important for the animal husbandry economy and their populations are rising steadily in Asia. China has the largest population of sheep and goats around the world [16]. Thus far, a number of studies have reported the infection of *G. duodenalis* in sheep and goats in China, with prevalences of 1.82–13.11% [13, 17–23] and 0.95–27.78% [17, 20, 24–30], respectively. Although asymptomatic infection of *G. duodenalis* commonly occurs in sheep and goats [31], an outbreak of giardiasis in a sheep farm located in central Italy had *G. duodenalis*-infected lambs showing malabsorption, weight loss, decrease of feed conversion ratio, malodorous and poorly formed greenish feces [32], causing significant economic loss to sheep on this farm. Assemblages A, B and E have been identified in sheep and goats in China, with assemblage E as the most frequently detected assemblage [3, 13, 30]. The identification of zoonotic assemblages A and B suggests that sheep and goats with *G. duodenalis* infections are likely to be the source of human giardiasis.

Black-boned sheep and black-boned goats, two important native breeds originated from Nanping county, Yunnan Province, southwestern China [33], are believed to have a medicinal function and nutritional value, as well as a health care value due to the great quantity of melanin in their tissues [33, 34]. However, to date, there are no studies on *G. duodenalis* infection in black-boned sheep and goats. Therefore, the objectives of the present study were to investigate the prevalence and assemblage distribution of *G. duodenalis* in black-boned sheep and goats, and to evaluate the zoonotic potential of *G. duodenalis* in these two native breeds using a high-resolution multilocus genotyping tool targeting three genes (*bg*, *gdh* and *tpi*) useful for studying intra-assemblage variation.

## Methods

### Sample collection

A total of 661 fecal samples were collected from five counties (Meishui, Shanshu, Shilin, Yongsheng and Nanping) in Yunnan Province, southwestern China in August 2017; of these, 325 fecal specimens were collected from black-boned sheep and 336 were collected from black-boned goats. Both male and female animals’ feces were collected, and these animals were divided into four age groups: 0–2 months; 3–6 months; 7–12 months; and > 12 months. Each fresh fecal specimen was randomly obtained from the rectum of each apparently healthy animal using a sterile disposal plastic glove. Approximately 5–20 g of feces were collected from each animal. The feces were marked with the sampling site, sex, age and breed immediately after collection, and kept on ice packs during transportation. All specimens were preserved in 2.5% potassium dichromate at 4 °C until genomic DNA extraction.

### Genomic DNA extraction

To remove the potassium dichromate, approximately 300 mg of each fecal specimen was washed by centrifugation at 13,000 × *g* with sterilized distilled water until the supernatant was clear before genomic DNA extraction. Then, the genomic DNA of each washed fecal specimen was extracted using E.Z.N.A^®^ Stool DNA kit (Omega Bio-tek Inc., Norcross, GA, USA), following the manufacturer’s instructions. The extracts of genomic DNA were stored at -20 °C for further PCR analysis.

### Nested PCR amplification

The prevalence of *G. duodenalis* in black-boned sheep and goats was determined by nested PCR targeting the *bg* gene using previously described primers and procedures [9, 35, 36]. Then, the *bg*-positive samples were analyzed by nested PCR at the *gdh* and *tpi* loci using previously described primers and procedures [9, 37]. Positive and negative controls were included in each PCR reaction. The secondary PCR products were screened by electrophoresis with 1% (*w*/*v*) agarose gels containing ethidium bromide.

### Sequencing and sequence analysis

All *G. duodenalis bg*-, *gdh*- and *tpi*-positive secondary PCR products were sent to Xi’an Sangon Biotech (Shanghai) Co., Ltd. for direct sequencing. Sequences obtained were first proofread with their DNA peak-form graph using Chromas v.2.6. Then, the checked sequences were amended by aligning with reference sequences downloaded from GenBank database using the software Clustal X v.1.83 [38] to identify *G. duodenalis* assemblages. Samples simultaneously successfully amplified at all three genetic loci (*bg*, *gdh* and *tpi*) were used to form MLGs to further reveal the genetic diversity. The nomenclature of novel sequence subtypes identified in the present study and those undesignated subtype sequences previously deposited in GenBank followed that of the former studies [13, 23].

### Statistical analysis

The differences in prevalence between different breeds, sexes, age groups and locations were calculated using a Chi-square test in SPSS 22.0 (SPSS Inc., Chicago, IL, USA). The differences were considered statistically significant when *P* < 0.05.

## Results

### Prevalence of *G. duodenalis* in black-boned sheep and goats

In the present study, of the 661 animal fecal specimens examined, 87 were *G. duodenalis bg*-positive, giving a total prevalence of 13.2%. Among the positive samples, 71 were from black-boned sheep [prevalence of 21.8% (71/325)] (Table 1), and 16 were from black-boned goats [prevalence of 4.8% (16/336)] (Table 1); a significant difference was observed between these two species (*χ*^2^ = 36.63, *df* = 1, *P* < 0.001). We also found that the occurrence of *G. duodenalis* decreased as age increased in these animals, with the highest prevalence found in animals < 2 months (54.3%, 19/35), followed by 3–6 months (37.3%, 19/51), 7–12 months (16.3%, 41/252) and > 12 months (2.5%, 8/323) (*χ*^2^ = 97.33, *df* = 3, *P* < 0.001).

**Table 1:** Distribution of *Giardia duodenalis* genotypes and subtypes in black-boned sheep and goats by counties in Yunnan Province, southwestern China

Location	No. positive/No. examined (%)		Genotype or subtype (*n*)					
	Black-boned sheep	Black-boned goats	Black-boned sheep			Black-boned goats		
			*bg*	*gdh*	*tpi*	*bg*	*gdh*	*tpi*
Meishui	0	10/61 (16.4)	0	0	0	E (4), A5 (6)	E (1), A1 (3)	E (3), A1 (5), A4 (1)
Shanshu	0	2/142 (1.4)	0	0	0	E (2)	E (2)	E (1), A1 (1)
Nanping	5/40 (12.5)	4/102 (3.9)	E (5)	E (1)	E (1)	E (4)	E (3)	E (1)
Yongsheng	18/89 (20.2)	0/31 (0)	E (18)	E (8)	E (9)	0	0	0
Shilin	48/196 (24.5)	0	E (47), A5 (1)	E (26)	E (31), A4 (2)	0	0	0
Total	71/325 (21.8)	16/336 (4.8)	E (70), A5 (1)	E (35)	E (41), A4 (2)	E (10), A5 (6)	E (6), A1 (3)	E (5), A1 (6), A4 (1)

Significant differences in prevalence of *G. duodenalis* were found among different age groups of both black-boned sheep (*χ*^2^ = 46.11, *df* = 3, *P *< 0.001) and goats (*χ*^2^ = 32.51, *df *= 3, *P *< 0.001). Of the 325 fecal samples collected from the four age groups of black-boned sheep, the highest prevalence of *G. duodenalis* was found for the 0–2-month group (59.4%, 19/32); however, *G. duodenalis* was not found in this age group of black-boned goats (0/3) due to the number of samples being too small. The highest prevalence of *G. duodenalis* was found in 3–6 month black-boned goats (24.1%, 7/29). In black-boned sheep, the lowest prevalence was found in sheep aged more than 12 months (5.7%, 7/123), while only one of 200 (0.5%) black-boned goats in this age group was found with *G. duodenalis* infection (Table 2).

**Table 2:** Distribution of *Giardia duodenalis* genotypes and subtypes in black-boned sheep and goats by age in Yunnan Province, southwestern China

Species	Age (month)	Sample size	No. positive (%)	Assemblage (*n*)
Black-boned sheep	0–2	32	19 (59.4)	E (19)
	3–6	22	12 (54.5)	E (11), A5 (1)
	7–12	148	33 (22.3)	E (33)
	> 12	123	7 (5.7)	E (7)
	Total	325	71 (21.8)	E (70), A5 (1)
Black-boned goat	0–2	3	0	–
	3–6	29	7 (24.1)	E (3), A5 (4)
	7–12	104	8 (7.7)	E (6), A5 (2)
	> 12	200	1 (0.5)	E (1)
	Total	336	16 (4.8)	E (10), A5 (6)

### Distribution of *G. duodenalis* assemblages

Sequence analysis of the *bg* gene indicated two assemblages (A and E) of *G. duodenalis* in both black-boned sheep and goats. Of the 71 *bg*-positive black-boned sheep samples, 70 isolates from three counties and four age groups belonged to the animal-specific assemblage E, while only one isolate from a 3-month sheep in Shilin county was identified as the zoonotic assemblage A. Ten isolates of assemblage E were detected in 16 *bg*-positive black-boned goat samples and all 6 assemblage A isolates were detected in Meishui County (Tables 1 and 2).

### Subtypes of assemblages A and E

Among the 325 samples of black-boned sheep examined, a total of 35 and 43 sequences were obtained by amplifying 71 *bg*-positive black-boned sheep samples at the *gdh* and *tpi* loci, respectively (Table 1). At the *bg* locus, 14 subtypes were generated by comparison of the 71 amended sequences obtained from black-boned sheep, with one and 13 identified to be assemblages A and E, respectively, including nine known subtypes (A5, E2, E3, E5, E6, E8, E9, E13 and E15) and five novel subtypes (named as E16, E17, E18, E20 and E22) (Table 3). Four subtypes were yielded by analysis of 35 amended sequences at the *gdh* locus. All of them represented assemblage E and three of them had been previously published with no subtype names; here we named them as E31, E32 and E33, respectively, while only one was identified as a novel subtype (named as E34) (Table 4). Sequence analysis of the 43 *tpi*-positive samples revealed one subtype of assemblage A and four subtypes of assemblage E in black-boned sheep. Among them, three subtypes had been previously reported, named as A4, E3 and E5 (Table 5). One sequence had been previously published without a subtype name; here we named it as E29, while the remaining novel subtype was named as E30 (Table 5).

**Table 3:** Sequence variation for *bg* locus of *Giardia duodenalis* assemblage E in black-boned sheep and goats in Yunnan Province, southwestern China

Subtype	Nucleotide at position										GenBank ID	No. positive	
	48	153	168	195	309	318	396	411	459	471		Black-boned sheep	Black-boned goats
Ref. sequence	T	A	C	G	G	C	T	C	C	C	KY633469		
E2	T	A	C	G	G	C	T	C	C	C	KY633469	1	1
E3	C	A	C	G	A	C	T	C	C	C	KT235937	1	0
E5	C	A	C	G	G	C	T	C	C	C	KY633466	21	4
E6	C	G	C	G	G	C	C	C	C	C	KY769090	1	0
E8	C	A	C	G	G	C	C	C	C	C	KY633465	11	0
E9	T	A	C	G	G	C	C	C	C	C	KY769091	4	0
E13	C	G	C	G	G	C	T	C	C	C	KY432853	10	1
E15	C	A	C	G	G	T	T	C	C	C	MH621341	2	0
E16	T	A	C	G	G	C	T	C	A	C	MK327168	7	2
E17	T	A	C	G	G	C	C	C	C	T	MK327176	7	0
E18	C	A	C	G	G	C	C	C	C	T	MK327177	2	0
E19	C	A	C	G	G	C	T	T	C	C	MK327159	0	1
E20	C	A	C	A	A	C	T	C	C	C	MK327170	1	0
E21	C	A	T	G	G	C	T	C	C	C	MK327160	0	1
E22	C	G	C	G	G	C	C	C	C	T	MK327165	2	0

**Table 4:** Sequence variation for *gdh* locus of *Giardia duodenalis* assemblage E in black-boned sheep and goats in Yunnan Province, southwestern China

Subtype	Nucleotide at position			GenBank ID	No. positive	
	156	231	321		Black-boned sheep	Black-boned goats
Ref. sequence	A	C	A	MH158464		
E31	G	C	G	MF034658	1	0
E32	A	C	A	MG976836	9	3
E33	G	C	A	MH158465	14	2
E34	A	T	A	MK327179	11	0

**Table 5:** Sequence variation for *tpi* locus of *Giardia duodenalis* assemblage E in black-boned sheep and goats in Yunnan Province, southwestern China

Subtype	Nucleotide at position						GenBank ID	No. positive	
	87	137	340	341	428	474		Black-boned sheep	Black-boned goats
Ref. sequence	G	A	A	T	G	G	KY769100		
E3	G	A	A	T	G	G	KY769100	32	3
E5	G	G	A	T	G	G	EF654686	2	0
E28	A	A	G	G	G	G	JX845451	0	2
E29	A	A	A	T	G	A	JQ951964	2	0
E30	A	A	G	G	A	G	MK327182	5	0

In black-boned goats, one subtype of assemblage A and six subtypes of assemblage E were identified at the *bg* locus, including four known subtypes (named as A5, E2, E5 and E13) and three novel subtypes (named as E16, E19 and E21) (Table 3). At the *gdh* locus, one known subtype (designated as A1), two previously published sequence with no subtype name (named as E32 and E33) were identified (Table 4). At the *tpi* locus two known assemblage A subtypes (A1 and A4), one known assemblage E subtype (E3) and one previously published sequence with no subtype name (named as E28) were identified (Table 5).

### Multilocus genotypes

In total, 33 samples were simultaneously amplified at all three intra-assemblage variation genetic loci, including 26 samples from black-boned sheep and seven from black-boned goats. Of the 26 black-boned sheep samples, sequences obtained from 24 samples belonged to assemblage E, forming 14 novel assemblage E MLGs (named as MLG-E1 to MLG-E8 and MLG-E10 to MLG-E15), and two samples were mixed infection with assemblages E and A (Table 6). In black-boned goats, three distinct assemblage E MLGs (named as MLG-E1, MLG-E2 and MLG-E9) were yielded from three samples and one assemblage A MLG (named as MLG-A1) was identified from another three samples; the remaining sample was a mixed infection with assemblages E and A (Table 6).

**Table 6:** Multilocus sequence genotyping of *Giardia duodenalis* in black-boned sheep and goats in Yunnan Province, southwestern China, based on *bg*, *tpi* and *gdh* genes

MLGs	Genotype			No. positive	
	*bg*	*tpi*	*gdh*	Black-boned sheep	Black-boned goats
MLG-E1	E5	E3	E32	4	1
MLG-E2	E5	E3	E33	1	1
MLG-E3	E5	E3	E34	1	0
MLG-E4	E5	E5	E32	2	0
MLG-E5	E8	E3	E34	3	0
MLG-E6	E9	E3	E33	2	0
MLG-E7	E13	E3	E33	2	0
MLG-E8	E13	E3	E34	1	0
MLG-E9	E16	E28	E33	0	1
MLG-E10	E16	E30	E33	3	0
MLG-E11	E17	E3	E33	1	0
MLG-E12	E17	E3	E34	1	0
MLG-E13	E17	E29	E33	1	0
MLG-E14	E18	E3	E33	1	0
MLG-E15	E22	E3	E33	1	0
MLG-A1	A5	A1	A1	0	3
Mixed 1 (A + E)	E8	A4	E33	1	0
Mixed 2 (A + E)	E13	A4	E34	1	0
Mixed 3 (A + E)	E19	A1	E32	0	1

In black-boned sheep, MLG-E1 was found in four samples from two counties. MLG-E4, MLG-E6 and MLG-E7 were each found in two samples from one county. MLG-E5 and MLG-E10 were each found in three samples from one county. The remaining eight assemblage E MLGs were observed in only one sample (Table 7). Most MLGs were found in Shilin county, three MLGs were seen in Yongsheng county and only one MLG was observed in Nanping county (Table 7). Most MLGs were observed in black-boned sheep of 7–12 months (7/15) and 0–2 months (6/15) (Table 8).

**Table 7:** Distribution of *Giardia duodenalis* MLGs in black-boned sheep and goats by county in Yunnan Province, southwestern China

Location	Black-boned sheep	Black-boned goats
Meishui	–	MLG-E9 (1), MLG-A1 (3)
Shanshu	–	–
Nanping	MLG-E15 (1)	MLG-E1 (1), MLG-E2 (1)
Yongsheng	MLG-E1 (1), MLG-E4 (2), MLG-E10 (3)	–
Shilin	MLG-E1 (3), MLG-E2 (1), MLG-E3 (1), MLG-E5 (3), MLG-E6 (2), MLG-E7 (2), MLG-E8 (1), MLG-E11 (1), MLG-E12 (1), MLG-E13 (1), MLG-E14 (1)	–

**Table 8:** Distribution of *Giardia duodenalis* MLGs in black-boned sheep and goats by age in Yunnan Province, southwestern China

Age (months)	Black-boned sheep	Black-boned goats
0–2	MLG-E3 (1), MLG-E4 (2), MLG-E5 (2), MLG-E6 (1), MLG-E7 (2), MLG-E12 (1)	–
3–6	MLG-E1 (2), MLG-E2 (1), MLG-E13 (1)	MLG-A1 (2), MLG-E9 (1)
7–12	MLG-E1 (2), MLG-E5 (1), MLG-E6 (1), MLG-E10 (3), MLG-E11 (1), MLG-E14 (1), MLG-E15 (1)	MLG-A1 (1), MLG-E1 (1)
> 12	MLG-E8 (1)	MLG-E2 (1)

In black-boned goats, both assemblage E MLGs and assemblage A MLGs were found. MLG-E1 and MLG-E2 were detected in 9-month-old and 2-year-old black-boned goats from Nanping county, respectively. MLG-E9 was found in a 3-month-old black-boned goat from Meishui county. All three samples of MLG-A1 were from Meishui county, two of them were collected from black-boned goats of 3–6 months and one was from a black-boned goat of the 7–12 months group (Tables 7 and 8).

## Discussion

Giardiasis, an important parasitic disease, is believed to be associated with water because infected hosts could shed *Giardia* cysts into environment which could cause water contamination [39]. Infection of people and animals through ingestion of water contaminated with infective cysts could lead to great public concern and enormous economic loss to animal husbandry [16]. One experimental study indicated that lambs infected with *G. duodenalis* showed a lower rate of weight gain, longer time to reach slaughter weight and lower carcass weight than control lambs [40].

In this investigation, *G. duodenalis* was detected in both black-boned sheep and goats, with respective prevalences of 21.8% (71/325) and 4.8% (16/336). The prevalence in black-boned sheep was higher than in other kinds of sheep previously reported in China (1.82–13.11%) [17–24], several parts of Australia (20.2%, 11.1%), Spain (11.1%, 19.2%), Ghana (12.9%), Italy (1.5%), Iran (19.8%) and Ethiopia (2.6%) [31, 41–47], but lower than that in Switzerland (29.8%), Canada (38%), USA (25.4%), Belgium (35.8%), Norway (26.8%), some areas of Australia (63.7%), Spain (42%, 89.2%), Greece (37.3%) and Brazil (34%) [48–56]. As black-boned sheep age, the *G. duodenalis* prevalence tends to decrease, with a significant difference being observed among different age groups. Our results were consistent with previous reports [17, 22, 23, 41, 49, 56, 57], suggesting that lambs could be more susceptible to *G. duodenalis* than adult sheep.

Similarly, the *G. duodenalis* prevalence in black-boned goats was slightly higher than that reported in some provinces of China [17, 20, 25–27] and Uganda (0) [58]. However, it was lower than most previous reports in goats worldwide [24, 26, 28–31, 42, 46, 48, 51, 55, 59–63]. There was a significant difference in the prevalence of *G. duodenalis* among different age groups, and the prevalence in black-boned goat kids (< 6 months) (21.9%, 7/32) was significantly higher than in black-boned goats over 6 months (3.0%, 9/304). A higher *G. duodenalis* prevalence in goat kids has also been observed in other studies [17, 29, 30, 48, 51, 55, 59, 64]. Many factors could contribute to these variations, e.g. the age of tested animals, sensitivity of diagnostic methods, loci amplified, sample sizes, climates and breeding methods. For example, the prevalence in 0–2-month black-boned sheep was 59.4% (19/32), while no *G. duodenalis* was detected in black-boned goats of this age group due to the limited number of sampled animals (*n* = 3).

Assemblage E was the most prevalent genotype in both black-boned sheep (70/71) and goats (10/16) in our study. Assemblage E was also reported as the predominate genotype in sheep and goats in other studies [13, 17, 23, 24, 29–31, 43, 45, 46, 50–54, 56, 57, 59, 61, 62]. Although assemblage E was generally believed to be animal-specific [65], sporadic reports from some areas with poor conditions in Egypt, Australia and Brazil suggest that it can also be found in humans [66–70], indicating its zoonotic transmission. In some studies, both zoonotic assemblages A and B were observed in sheep and goats [17, 30, 44, 52, 61–63]. However, in our study, only one black-boned sheep and six black-boned goats were infected with *G. duodenalis* assemblage A. Interestingly, genomic analysis of assemblages A, B and E showed that the nucleotide similarity between assemblages A and E is much higher than that between assemblages A and B [71, 72]. These results suggest that infected black-boned sheep and goats have the opportunity to transmit *G. duodenalis* to humans.

To further reveal the genetic variations in assemblages A and E, another two genetic loci (*gdh* and *tpi*) of 87 *bg*-positive samples were analyzed by the multilocus genotyping tool with high resolution [9]. A total of four assemblage E subtypes at the *gdh* locus, four assemblage E subtypes and one assemblage A subtype at the *tpi* locus were identified in black-boned sheep. Two assemblage E subtypes and one assemblage A subtype at the *gdh* locus, and two assemblage E subtypes and two assemblage A subtypes at the *tpi* locus were observed in black-boned goats (Tables 4 and 5). In addition, three samples had different assemblages at the three loci (Table 6). Fifteen MLGs of assemblage E and one MLG of assemblage A were yielded in 30 samples amplified successfully at all three loci with the same assemblage genotype. These findings suggest that there is a high subtype diversity and genetic variation in assemblages A and E. In addition, a higher prevalence of *G. duodenalis* assemblage A in black-boned goats (62.5%, 10/16) and the presence of the assemblage A MLGs suggest that the black-boned goats has higher potential in transmitting zoonotic *G. duodenalis* than the black-boned sheep.

## Conclusions

The present study revealed the presence and identified assemblages of *G. duodenalis* in black-boned sheep and goats in Yunnan Province, southwestern China. Assemblages A and E were found in both black-boned sheep and goats, with assemblage E being the most prevalent in these two native species. The presence of zoonotic assemblage A in black-boned sheep and goats suggests their zoonotic potential. Multilocus genotyping at *bg*, *tpi* and *gdh* loci formed 14 novel assemblage E MLGs in black-boned sheep and four novel MLGs (three assemblage E MLGs and one assemblage A MLG) in black-boned goats. These results provide baseline data for preventing and controlling *G. duodenalis* infection in black-boned sheep and goats as well as humans.

## Abbreviations

*Bg*: β-giardin
*Gdh*: glutamate dehydrogenase
MLGs: multilocus genotypes
*Tpi*: triosephosphate isomerase
